# Serum Calretinin and Genetic Variability as a Prognostic and Predictive Factor in Malignant Mesothelioma

**DOI:** 10.3390/ijms25010190

**Published:** 2023-12-22

**Authors:** Cita Zupanc, Alenka Franko, Danijela Štrbac, Viljem Kovač, Vita Dolžan, Katja Goričar

**Affiliations:** 1 Military Medical Unit-Slovenian Army, 1000 Ljubljana, Slovenia; cita.zupanc@mors.si; 2 Faculty of Medicine, University of Ljubljana, 1000 Ljubljana, Slovenia; alenka.franko@kclj.si (A.F.); dstrbac@onko-i.si (D.Š.); vkovac@onko-i.si (V.K.); 3 Clinical Institute of Occupational Medicine, University Medical Centre Ljubljana, 1000 Ljubljana, Slovenia; 4 Institute of Oncology Ljubljana, 1000 Ljubljana, Slovenia; 5 Pharmacogenetics Laboratory, Institute of Biochemistry and Molecular Genetics, Faculty of Medicine, University of Ljubljana, 1000 Ljubljana, Slovenia; vita.dolzan@mf.uni-lj.si

**Keywords:** malignant mesothelioma, calretinin, CALB2, biomarker, polymorphism

## Abstract

Calretinin is a promising diagnostic biomarker for malignant mesothelioma (MM), but less is known about its prognostic role. Our aim was to evaluate the association between serum calretinin concentration or genetic factors and the survival or outcome of cisplatin-based chemotherapy in MM. Our study included 265 MM patients. Serum calretinin concentration was determined using ELISA. Patients were genotyped for seven polymorphisms in *CALB2*, *E2F2*, *MIR335*, *NRF1*, and *SEPTIN7* using competitive allele-specific PCR. Nonparametric tests, logistic regression, and survival analysis were used for statistical analysis. Higher serum calretinin concentration was associated with shorter progression-free (PFS) (HR = 1.18 (1.02–1.37), *p* = 0.023) and overall survival (OS) (HR = 1.20 (1.03–1.41), *p* = 0.023), but the association was not significant after adjusting for clinical factors (HR = 1.05 (0.85–1.31), *p* = 0.653 and HR = 1.06 (0.84–1.34), *p* = 0.613, respectively). *SEPTIN7* rs3801339 and *MIR335* rs3807348 were associated with survival even after adjustment (HR = 1.76 (1.17–2.64), *p* = 0.007 and HR = 0.65 (0.45–0.95), *p* = 0.028, respectively). Calretinin concentration was higher in patients who progressed after treatment with cisplatin-based chemotherapy (1.68 vs. 0.45 ng/mL, *p* = 0.001). Calretinin concentration above 0.89 ng/mL was associated with shorter PFS and OS from the start of chemotherapy (HR = 1.88 (1.28–2.77), *p* = 0.001 and HR = 1.91 (1.22–2.97), *p* = 0.004, respectively), even after adjusting for clinical factors (*p* < 0.05). *MIR335* rs3807348 was associated with a better response to chemotherapy (OR = 2.69 (1.17–6.18), *p* = 0.020). We showed that serum calretinin is associated with survival and chemotherapy treatment outcomes in MM and could serve as a predictive biomarker.

## 1. Introduction

Malignant mesothelioma (MM) is a rare cancer and is frequently associated with asbestos exposure [1]. It most often appears on the pleura and the peritoneum, but it can also occur in other parts of the body, such as the pericardium and tunica vaginalis. MM is very aggressive and has a poor prognosis, with a 5-year overall survival (OS) below 10% [2]. Currently, MM treatment consists of chemotherapy, surgery, and radiation. The introduction of chemotherapy significantly improved OS, and several trials confirmed the benefit of systemic treatment with drug combinations, such as doublets of cisplatin, of pemetrexed or gemcitabine, but standard treatment approaches have not changed much since the introduction of pemetrexed/cisplatin chemotherapy in 2004 [1,3]. In recent years, immunotherapy also emerged as a potential treatment option, but it is mostly not used as a first-line treatment [1,4]. Due to the limitations of individual treatment regimens, several new treatment targets and approaches are currently being investigated in preclinical studies and clinical trials [1,4,5], but despite recent advances, long-term benefits for MM patients are often lacking [6].

Minimally invasive serum or plasma biomarkers are one of the factors that could improve the prognosis of patients with MM. Diagnostic biomarkers, such as commonly studied mesothelin and fibulin, could allow an earlier diagnosis of MM and, therefore, enable detection of the disease in earlier stages, leading to a better prognosis [7,8,9,10]. Predictive or prognostic biomarkers, such as mesothelin or high-mobility group box protein 1 (HMGB1), could help guide treatment selection and enable a more personalized treatment, leading to better treatment outcomes [7,8,9,10]. Mesothelin and fibulin are increased in MM but have limited sensitivity or specificity [7,8,9]. Therefore, studies have tried to establish new biomarkers or combinations of biomarkers that could help diagnose MM or predict its prognosis. Among protein biomarkers, the highest predictive value was reported for mesothelin, fibulin-3, osteopontin, and HMGB1 [10]. On the mRNA level, more than 50 overexpressed genes were reported in MM that could serve as diagnostic, predictive, or prognostic markers, or even therapeutic targets, including *MSLN*, encoding for mesothelin, *EFEMP1*, encoding for fibulin 3, *BIRC5*, encoding for survivin and *CALB2*, encoding for calretinin [11]. Among other biomarker types, most studies focused on microRNAs (miRNAs), non-coding RNA that have an important role in the post-transcriptional regulation of gene expression [10] and can be detected in various bodily fluids, as well as in extracellular vesicles [12]. MiRNAs might play an oncogenic or a tumor-suppressive role in multiple cancers and have already been proposed as both biomarkers and therapeutic targets in MM [1]. For example, an altered expression of miR-103-3p, miR-126-3p, and miR-625-3p was reported in MM, and miR-126-3p and miR-625-3p were also associated with MM prognosis [10,13]. However, even though several biomarkers were reported, none have resulted in a clinically validated test, partly due to the heterogeneity among studies regarding control groups, cutoff values, and biological fluids [10].

One of the clinically validated immunohistological MM biomarkers is calretinin, a calcium-binding protein that plays an important role in neurons and is also expressed on the surface of mesothelial cells during MM tumorigenesis [14]. Calretinin can activate the focal adhesion kinase (FAK) signaling pathway and promote invasion and epithelial-to-mesenchymal transition [15]. Calretinin is a useful tissue diagnostic biomarker that can distinguish MM from other asbestos-related diseases and is commonly used in immunohistochemical diagnosis of MM. It can also be found in serum, plasma, and pleural effusions of MM patients, as well as extracellular vesicles (EVs) derived from MM cell lines [16,17]. Calretinin plasma or serum concentration is higher in patients with MM than healthy people or patients with benign asbestos-related diseases [16,18,19,20] and it could, therefore, also contribute to an earlier non-invasive diagnosis of MM as a circulating biomarker [18].

Less is known about the association of calretinin with survival and treatment outcome, even though the expression of calretinin in tumor tissue was previously associated with the survival of MM patients. Higher tissue calretinin expression was associated with the epithelioid histological type and was an independent predictor of better survival [21,22,23]. Previous studies also suggest that calretinin might be associated with a response to cisplatin-based chemotherapy, which is commonly used in MM. In MM cell lines, calretinin downregulation was associated with resistance to cisplatin through the FAK and Wnt signaling pathways [15]. In ovarian cancer patients treated with cisplatin, higher serum calretinin at diagnosis or a relative serum calretinin increase during treatment were both independent predictors of platinum resistance, as well as survival [24]. However, the predictive and prognostic value of soluble calretinin has not been previously investigated in MM.

Different clinical and tumor characteristics can affect the concentration of soluble calretinin, such as sex and histological type [25,26]. Calretinin expression is also affected by different molecular factors: transcription factors, e.g., septin 7, E2F transcription factor 2 (E2F2), and nuclear respiratory factor 1 (NRF-1), as well as miRNAs, e.g., miR-335-5p [14,27,28], but the regulation of calretinin in different tissues is still not well understood [14]. Clinical characteristics, genetic variability, and molecular factors that can affect calretinin expression could potentially be used to improve the predictive value of soluble calretinin as a biomarker in MM.

The aim of our study was, therefore, to investigate the association between serum calretinin concentration or genetic factors associated with calretinin expression and the survival of MM patients or the outcomes of cisplatin-based chemotherapy in MM.

## 2. Results

### 2.1. Patients’ Characteristics and Genotype Frequencies

In total, 265 MM patients were included in this study, and among them, 239 were treated with cisplatin-based chemotherapy. The clinical characteristics of all MM patients and MM patients treated with cisplatin-based chemotherapy are presented in Table 1. In the whole group, 197 (74.3%) patients were male, 235 (88.7%) had pleural MM, and 204 (77.0%) had the epithelioid histological type. Median progression-free survival (PFS) from diagnosis was 10.0 (6.3–16.2) months and median OS from diagnosis was 19.1 (10.0–29.4) months, with a follow-up from diagnosis of 45.5 (22.8–78.7) months. Serum calretinin concentration was determined at diagnosis for 146 patients and was 0.52 (0.22–1.43) ng/mL.

All patients were genotyped for seven single nucleotide polymorphisms (SNPs) in *CALB2*, *E2F2*, *MIR335*, *NRF1*, and *SEPTIN7* genes. The genotype frequencies of the studied SNPs are presented in Table S1. Variant allele frequencies ranged between 12 and 56%. All SNPs met the genotyping quality control criteria. All genotype frequencies were consistent with the expected distribution in the population under the Hardy–Weinberg equilibrium (HWE) (all *p* > 0.05).

### 2.2. Association of Calretinin with Progression-Free Survival and Overall Survival from Diagnosis

Among clinical factors, asbestos exposure, smoking, weight loss, C-reactive protein, and histological type were significantly associated with PFS from diagnosis (all *p* < 0.05), while age, asbestos exposure, smoking, C-reactive protein, and histological type were significantly associated with OS from diagnosis (all *p* < 0.05).

Higher serum calretinin concentration was associated with shorter PFS (HR = 1.18, 95% CI = 1.02–1.37, *p* = 0.023) and shorter OS in MM patients (HR = 1.20, 95% CI = 1.03–1.41, *p* = 0.023). However, after adjusting for the significant clinical factors, the association was no longer statistically significant (HR = 1.05, 95% CI = 0.85–1.31, *p* = 0.653 and HR = 1.06, 95% CI = 0.84–1.34, *p* = 0.613, respectively). If patients were divided into two groups based on median calretinin concentration, PFS was slightly shorter if serum calretinin concentration was above the median (8.3 (5.7–14.2) compared to 10.0 (6.8–14.5) months), but the difference was not significant in univariable (HR = 1.25, 95% CI = 0.89–1.77, *p* = 0.205) or multivariable analysis (HR = 0.95, 95% CI = 0.63–1.43, *p* = 0.816). Similarly, OS was shorter if serum calretinin concentration was above the median (14.9 (9.9–23.9) compared to 20.7 (11.3–43.4) months), but the difference did not reach statistical significance in univariable (HR = 1.39, 95% CI = 0.94–2.05, *p* = 0.104) or multivariable analysis (HR = 0.97, 95% CI = 0.60–1.55, *p* = 0.884).

The association of investigated SNPs with the survival of MM patients from diagnosis is presented in Table 2. Among genetic factors, *SEPTIN7* rs3801339 was associated with slightly shorter PFS in carriers of two polymorphic alleles (HR = 1.47, 95% CI = 1.02–2.13, *p* = 0.039), even after adjusting for clinical factors (HR = 1.76, 95% CI = 1.17–2.64, *p* = 0.007) (Figure S1). In multivariable analysis, the association was also significant in the dominant model (*p* = 0.046, Table 2). Heterozygotes for the *MIR335* rs3807348 polymorphism had longer OS compared to the carriers of two reference alleles (HR = 0.69, 95% CI = 0.49–0.98, *p* = 0.038), even after adjusting for clinical factors (HR = 0.65, 95% CI = 0.45–0.95, *p* = 0.028).

### 2.3. Association of Calretinin with the Outcome of Cisplatin-Based Chemotherapy

Among patients treated with cisplatin-based chemotherapy, 154 (64.4%) patients received a combination of gemcitabine and cisplatin, while 85 (35.6%) patients received a combination of pemetrexed and cisplatin. Regarding treatment outcome, eight (3.5%) patients had a complete response, 72 (31.2%) patients had a partial response, 112 (48.5%) had stable disease, and 39 (16.9%) experienced disease progression.

**Figure 1:**
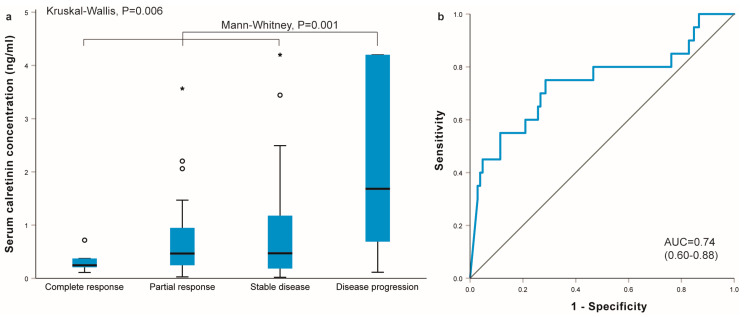
(**a**) Serum calretinin concentration and response to chemotherapy in malignant mesothelioma patients; (**b**) ROC curve analysis for the prediction of disease progression based on serum calretinin concentration at diagnosis. AUC, area under the curve. Circles represent outliers and stars (*) represent extreme outliers.

Serum calretinin concentration differed in MM patients with different treatment outcomes. The highest calretinin concentrations were observed in patients with disease progression after chemotherapy and the lowest were observed in patients who responded completely to chemotherapy (*p* = 0.006, Figure 1a). Calretinin concentration was statistically significantly higher in patients who progressed after the completion of chemotherapy compared to others (1.68 (0.58–4.20) ng/mL versus 0.45 (0.21–1.07) ng/mL, *p* = 0.001). Calretinin concentration at diagnosis was a good predictor of disease progression (area under the curve 0.74 (0.60–0.88); *p* = 0.001) (Figure 1b). At a cutoff of 0.89 ng/mL, the sensitivity for predicting disease progression was 0.750 and the specificity was 0.714.

The association of serum calretinin concentration with disease control rate is presented in Table 3 and Table S2. Patients with calretinin concentration above the median were more likely to experience disease progression (OR = 3.56, 95% CI = 1.21–10.52, *p* = 0.021), but the difference was no longer significant after adjusting for CRP and chemotherapy type (*p* = 0.168). The difference was even more pronounced if patients were divided based on the cutoff value determined in ROC curve analysis: patients with a calretinin concentration above 0.89 ng/mL were much more likely to experience disease progression (OR = 7.50, 95% CI = 2.50–22.47, *p* < 0.001), even after adjusting for clinical factors (*p* = 0.016, Table S2).

When evaluating the association with response rate, patients with higher serum calretinin concentration were less likely to achieve good treatment response (OR = 0.61, 95% CI = 0.40–0.93, *p* = 0.021), but the association was no longer significant after adjusting for smoking, CRP, and weight loss (OR = 0.77, 95% CI = 0.48–1.24, *p* = 0.278). Additionally, serum calretinin above the median or above 0.89 ng/mL was not significantly associated with the response rate in univariable (*p* = 0.315 and *p* = 0.632, respectively) or multivariable analysis (*p* = 0.948 and *p* = 0.695, respectively).

When analyzing the effect of selected polymorphisms on the treatment response rate (Table 4), carriers of at least one *MIR335* rs3807348 polymorphic allele had a significantly better response (OR = 2.41, 95% CI = 1.19–4.87, *p* = 0.015), even after adjusting for clinical factors (OR = 2.69, 95% CI = 1.17–6.18, *p* = 0.020). Other investigated polymorphisms were not significantly associated with response rate (all *p* > 0.05).

### 2.4. Association of Calretinin with Progression-Free Survival and Overall Survival from the Beginning of Chemotherapy

**Figure 2:**
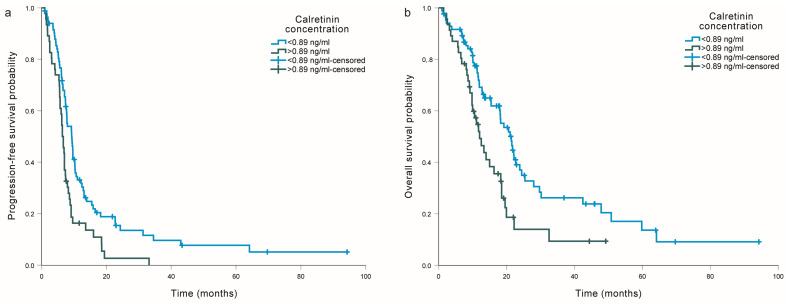
Association of serum calretinin concentration with (**a**) progression-free survival and (**b**) overall survival from the beginning of chemotherapy in malignant mesothelioma patients treated with cisplatin-based chemotherapy.

Serum calretinin concentration was also associated with survival from the beginning of chemotherapy in MM patients treated with cisplatin-based chemotherapy (Table 3 and Table S2). Higher serum calretinin concentration was associated with worse PFS (*p* = 0.010) and worse OS (*p* = 0.013) in univariable analysis, but not after adjusting for clinical factors (*p* = 0.206 and *p* = 0.378, respectively). Patients with a calretinin concentration above 0.89 ng/mL had significantly shorter PFS (HR = 1.88, 95% CI = 1.28–2.77, *p* = 0.001; Figure 2a) and shorter OS (HR = 1.91, 95% CI = 1.22–2.97, *p* = 0.004, Figure 2b). This association remained statistically significant, even after adjusting for clinical factors (*p* = 0.006 and 0.049, respectively).

None of the studied polymorphisms were significantly associated with PFS or OS in this group of patients, either in univariable or multivariable analysis (Table S3).

### 2.5. In Silico Analysis of Tumor Tissue mRNA Expression

We also evaluated the mRNA expression of investigated protein-coding genes in TCGA MESO samples using the online tool GEPIA2. There were several correlations between the expression of different genes in the MM tumor tissue. We observed significant negative correlations between *CALB2* and *E2F2* (ρ = −0.26, *p* = 0.017), as well as between *CALB2* and *SEPTIN7* (ρ = −0.29, *p* = 0.007) expression, while the correlation between *CALB2* and *NRF1* (ρ = −0.20, *p* = 0.058) did not reach statistical significance. On the other hand, positive correlations were observed between *E2F2* and *NRF1* (ρ = 0.32, *p* = 0.002), *E2F2* and *SEPTIN7* (ρ = 0.28, *p* = 0.009), and *NRF1* and *SEPTIN7* expression (ρ = 0.28, *p* < 0.001).

**Figure 3:**
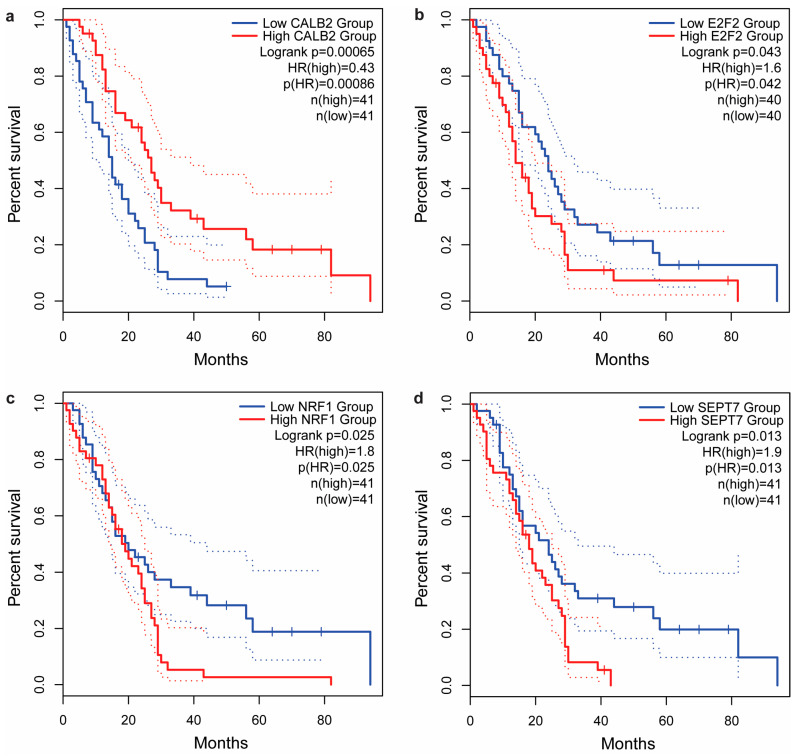
Association of the tumor tissue gene expression of the investigated genes with overall survival based on The Cancer Genome Atlas mesothelioma (TCGA MESO) data evaluated using the GEPIA2 online tool: (**a**) *CALB2*, (**b**) *E2F2*, (**c**) *NRF1*, (**d**) *SEPTIN7*. Dotted lines represent 95% confidence intervals.

The association of gene expression with the survival of MM patients showed that disease-free survival (DFS) was only statistically significantly affected by *SEPTIN7* (HR = 1.9, *p* = 0.03), while no significant differences were observed for *CALB2* (HR = 0.80, *p* = 0.42), *E2F2* (HR = 1.6, *p* = 0.099), and *NRF1* (HR = 1.7, *p* = 0.065). On the other hand, all four genes were significantly associated with OS of MM patients: patients with high *CALB2* expression had longer survival than patients with low *CALB2* expression (HR = 0.43, *p* < 0.001, Figure 3a), while patients with higher *E2F2* (HR = 1.6, *p* = 0.042, Figure 3b), *NRF1* (HR = 1.8, *p* = 0.025, Figure 3c), or *SEPTIN7* (HR = 1.9, *p* = 0.013, Figure 3d) expression had shorter OS.

## 3. Discussion

In the present study, we evaluated the prognostic and predictive role of serum calretinin concentration in patients with MM. We also assessed if genetic polymorphisms in the *CALB2* gene or in genes coding for miRNAs and transcription factors that regulate calretinin expression can serve as independent prognostic factors in MM. In the whole patient group, higher serum calretinin concentration was associated with shorter PFS and shorter OS, but the association was no longer significant after adjusting for clinical factors, indicating that calretinin was not an independent prognostic factor in our patients with MM. On the other hand, calretinin was an important predictive biomarker in MM patients treated with cisplatin-based chemotherapy, even in multivariable analysis: serum calretinin concentration was significantly higher in patients who had disease progression during or after the completion of chemotherapy and was associated with worse PFS and OS. Among genetic factors, *SEPTIN7* rs3801339 was associated with shorter PFS, and *MIR335* rs3807348 was associated with longer OS and better response to cisplatin-based chemotherapy.

In our study, serum calretinin concentration was associated with shorter PFS and OS from diagnosis, regardless of the treatment regimen, but after adjusting for clinical factors, the association was no longer statistically significant, suggesting that serum calretinin is not an independent prognostic factor in MM. As MM is commonly treated with cisplatin-based chemotherapy, we also determined the association of calretinin with the outcome of cisplatin-based chemotherapy and survival from the start of chemotherapy. We demonstrated that serum calretinin concentration was higher in patients with a worse treatment outcome and shorter PFS and OS from the beginning of chemotherapy, even after adjusting for clinical factors, suggesting that it could serve as an independent predictive biomarker in MM patients treated with cisplatin-based chemotherapy. A calretinin concentration above 0.89 ng/mL was a good predictor of disease control rate and survival, but not response rate, suggesting that it is associated more with disease progression. This concentration is much higher than the concentration of 0.32 ng/mL, which could be used as a diagnostic biomarker to discriminate between MM patients and other asbestos-exposed subjects based on our previous results [18].

So far, none of the previous studies investigated the association of soluble calretinin with prognosis or treatment outcome in MM. Consistent with our results, higher serum calretinin concentration at diagnosis, as well as its dynamics during chemotherapy, were independent predictors of poor PFS and OS, as well as chemotherapy resistance, in ovarian cancer patients treated with cisplatin-based chemotherapy [24]. Additional validation studies are needed to better evaluate the association between calretinin and cisplatin and determine whether serum calretinin could serve as a predictive biomarker of disease progression in cancer patients treated with cisplatin in clinical practice.

Contrary to our results and results in ovarian cancer showing that higher serum calretinin concentration is associated with shorter survival [24], previous studies reported that the higher expression of calretinin in MM tumor tissue was independently associated with longer survival, even after adjusting for significant clinical parameters, such as histological subtype or age [21,22,23]. Therefore, we also evaluated mRNA expression in MM tumor tissue using in silico analysis of publicly available databases. Consistent with previous studies, higher calretinin mRNA expression was associated with longer OS in the TCGA MESO cohort. So far, the association between tissue calretinin expression and soluble calretinin in MM was only investigated in one study: patients with calretinin-positive tumors had higher plasma calretinin [29], and, therefore, a similar prognostic role in tissue and blood could be expected. However, the positive correlation of calretinin expression in tissue and plasma could be attributed to the fact that calretinin is more often detected in epithelioid and biphasic MM compared to the sarcomatoid subtype in both tissue and plasma or serum of MM patients [18,20,30,31]. As patients with sarcomatoid MM still have much shorter survival [32], this could explain the observed differences regarding the association of calretinin with survival. However, a more detailed comparison would be needed to determine the different prognostic roles of calretinin in tissue and blood samples.

In our bioinformatic analysis, we also evaluated tissue mRNA expression for other protein-coding genes investigated in our study. For *E2F2*, *NRF1*, and *SEPTIN7*, MM patients with lower mRNA expression had longer OS. Additionally, significant correlations were observed between the mRNA expression of *CALB2* and genes involved in calretinin regulations in MM tumor tissue, as well as among the investigated regulating genes, further confirming that *E2F2*, *NRF1*, and *SEPTIN7* could act as regulators of calretinin expression as previously proposed [14,27]. Further studies are needed to assess whether a combination of these genes might serve as a potential biomarker in MM.

In the genetic part of our study, we also investigated the association of several different SNPs in *CALB2* and its regulating genes with survival and treatment response in MM. We found that carriers of two polymorphic *SEPTIN7* rs3801339 alleles had shorter PFS from diagnosis, even after adjusting for clinical factors. Septin 7 is a GTP-binding protein that plays an important role in various cellular processes [14,33]. Its binding to the *CALB2* promoter region resulted in decreased calretinin expression in MM cell lines [14]. We also observed a negative correlation between *SEPTIN7* and *CALB2* expression in TCGA MESO tissue samples. Septin 7 can also contribute to cancer development [33,34] and it was previously implicated in cell migration and invasion in breast cancer cell lines [35]. Similar to our results, another polymorphism in this gene, rs1143149, was previously associated with shorter survival in non-small cell lung cancer patients who were long-term former smokers [33]. On the other hand, *SEPTIN7* rs3801339 was not significantly associated with MM susceptibility or with serum calretinin concentration in our previous study [36]. Additional studies are needed to better evaluate the association of septin 7 and its genetic variability with both calretinin and cancer prognosis.

In the present study, the polymorphic *MIR335* rs3807348 allele was associated with longer OS from diagnosis, as well as a better response to cisplatin-based chemotherapy, even after adjusting for clinical factors. In our previous study, this allele was also associated with higher serum calretinin concentration [36]. MiR-335 can influence apoptosis, proliferation, migration, and invasion of cancer cells through various target genes involved in different signaling pathways, including *CALB2*, and is often dysregulated in different cancer types [37]. In most cancer types, miR-335 is downregulated and acts as a tumor suppressor, but some studies reported that it is upregulated and acts as an oncogene in specific cancers [37]. Some studies also reported that it could function as a prognostic biomarker; however, both lower and higher miR-335 expression was associated with worse prognosis in different cancer types [37,38]. In MM, miR-335 expression was only evaluated in a very small study, where increased expression was reported in the serum of MM patients [39]. Interestingly, miR-335 was also associated with response to chemotherapy, including cisplatin-based chemotherapy [37]. In ovarian cancer, increased miR-335 expression was associated with increased sensitivity to cisplatin through its target gene *BCL2L2*, an anti-apoptotic protein increased in several cancer types [40]. However, the mechanism of action of miR-335 is complex and associated with many cellular processes. Additionally, the role of *MIR335* genetic variability is not well established. Further studies are, therefore, needed to improve the understanding of miR-335’s association with calretinin and its role in MM and cancer in general.

Other investigated SNPs in *CALB2*, *E2F2*, and *NRF1* genes were not associated with survival or response to cisplatin-based chemotherapy, even though we have previously shown that they could contribute to the interindividual variability observed for serum calretinin concentration in MM patients or asbestos-exposed subjects [36], suggesting that the genetic variability in these genes does not play an important role as a prognostic or predictive biomarker in MM.

This is the first study to show that serum calretinin and genetic factors associated with calretinin expression might play a role in prognosis and treatment response in MM, but this study also has some limitations. In our study, serum calretinin concentration was only available for a subgroup of patients and we did not have tissue samples available for comparison of calretinin expression in tumor samples. To overcome this, we used in silico analysis in MM samples included in publicly available databases. As expression data for miR-335 are not available in the GEPIA2 database, this part of the analysis was only performed for protein-coding genes. On the other hand, we evaluated the role of genetic variability in a large MM cohort. Still, other genetic factors could also influence calretinin expression. All MM patients were also treated in the same institution according to the same protocols. In the future, further large studies in this field and validation of these results in an independent population are needed. Additionally, evaluation of the mRNA and protein expression of investigated genes and the miRNA expression of investigated miRNAs in different biological samples could provide new insights about the processes involved in MM and its prognosis.

So far, several circulating biomarkers have been identified in MM, especially diagnostic, but also prognostic. Still, none of them have entered routine clinical use yet, and further validation studies and studies focusing on new combinations of biomarkers are needed. The association of calretinin with response to cisplatin reported in our study is also important because many new treatment options are being investigated in MM, such as immunotherapy and targeted therapy. With the help of new biomarkers and genetic factors, we could identify patients who are expected to respond poorly to standard forms of chemotherapy and would benefit more from other treatment options.

## 4. Materials and Methods

### 4.1. Study Population

Our retrospective study included patients with histologically confirmed pleural or peritoneal MM treated at the Institute of Oncology Ljubljana between November 2001 and March 2019.

For all patients, data on demographic and clinical characteristics were obtained from medical records or an interview. Asbestos exposure was defined as known occupational or environmental exposure. The stage of MM was determined using the TNM staging system for pleural MM. The performance status of MM patients was determined using Eastern Cooperative Oncology Group (ECOG) scores.

All participants provided written informed consent. This study was approved by the National Medical Ethics Committee of the Republic of Slovenia (31/07/04, 39/04/06, and 41/02/09) and was carried out according to the Declaration of Helsinki.

### 4.2. Treatment Response and Survival Assessment

Tumor response was evaluated using modified Response Evaluation Criteria in Solid Tumors (RECISTs) [41]. The response rate was defined as the percentage of patients that achieved complete or partial response, while the disease control rate was defined as the percentage of patients that achieved complete response, partial response, or stable disease.

In survival analysis, PFS and OS were evaluated. PFS was defined as the time from diagnosis (for all patients) or from the beginning of chemotherapy (for patients with systemic treatment) to disease progression, and OS was defined as the time from diagnosis or from the beginning of chemotherapy to death from any cause.

### 4.3. Single-Nucleotide Polymorphism (SNP) Selection, DNA Extraction, and Genotyping

Using bioinformatic analysis, we previously identified common SNPs that could affect calretinin expression within the *CALB2* gene and its regulating transcription factors and miRNAs [36]. We selected three SNPs in 5′ and 3′ UTR of the *CALB2* gene (rs1862818, rs889704, rs8063760), three SNPs in genes coding for transcription factors (*E2F2* rs2075995, *NRF1* rs13241028, *SEPTIN7* rs3801339) and one SNP in genes coding for miRNAs associated with calretinin expression (*MIR335* rs3807348).

We used the Qiagen FlexiGene Kit (Qiagen, Hilden, Germany) to extract genomic DNA from peripheral venous blood samples. All SNPs were genotyped using competitive allele-specific PCR following the manufacturer’s instructions (KASP assays, LGC Biosearch Technologies, Hoddesdon, UK). For all SNPs, 15% of the samples were genotyped in duplicates. Genotyping quality control criteria were 100% duplicate call rate and 95% SNP-wise call rate.

### 4.4. Measurement of Serum Calretinin

Serum samples were collected at diagnosis, prepared within 6 h of blood collection, and stored at −20 °C until analysis. Serum calretinin concentrations were determined using a commercially available enzyme-linked immunosorbent calretinin ELISA assay (DLD Diagnostika GmbH, Hamburg, Germany) according to the manufacturer’s instructions, as previously described [18,20,42]. The manufacturer reported the intra-assay coefficient of variation as 8.1–6.6% and the inter-assay coefficient of variation as 10.4–10.0%.

### 4.5. Bioinformatic Analysis: Tissue Gene Expression

To analyze the expression of calretinin and associated genes in MM tissue samples, we used the Gene Expression Profiling Interactive Analysis (GEPIA) 2 tool, an interactive web server for analyzing the RNA sequencing expression data of tumors and normal samples from The Cancer Genome Atlas (TCGA). We used GEPIA2 to assess the correlations between the expression of different protein-coding genes in MM and evaluate the association of the investigated genes with survival in MM in the TCGA mesothelioma tumor samples (TCGA MESO study) [43]. Expression above the median was classified as high expression, while expression below the median was classified as low expression.

### 4.6. Statistical Analysis

Continuous and categorical variables were described using a median with interquartile range and frequencies, respectively. For all investigated SNPs, both additive and dominant models were used in the analysis. A chi-square test was used to evaluate deviation from HWE. Nonparametric Mann–Whitney or Kruskal–Wallis tests were used to compare the distribution of continuous variables. Spearman’s rho correlation coefficient (ρ) was used to evaluate correlations between continuous variables. Receiver operating characteristic (ROC) curve analysis was used to determine the area under the curve (AUC), sensitivity, and specificity. Cutoff values were determined as the values with the highest sum of sensitivity and specificity. Univariable and multivariable logistic regression was used to evaluate the association with response to chemotherapy to determine odds ratios (ORs) and 95% confidence intervals (CIs). In survival analysis, Kaplan–Meier analysis was used to calculate the median survival or follow-up time and analysis of GEPIA survival data, while univariable and multivariable Cox regression was used to calculate the hazard ratios (HRs) with 95% CIs. Patients without progression or death at the time of the analysis were censored at the date of the last follow-up. Clinical parameters used for adjustment in multivariable analysis were selected using stepwise forward conditional selection. To determine the median survival in patients with different serum calretinin concentrations, patients were divided into two groups according to median concentration or cutoff values determined using ROC curve analysis. All statistical tests were two-sided, and the level of significance was set at 0.05. The statistical analyses were carried out using IBM SPSS Statistics version 27.0 (IBM Corporation, Armonk, NY, USA).

## 5. Conclusions

In conclusion, our study showed that higher serum calretinin concentration was associated with shorter PFS and OS in MM, even though it was not an independent prognostic factor in our study. *SEPTIN7* rs3801339 and *MIR335* rs3807348 were associated with survival among genetic factors. Additionally, serum calretinin concentration could serve as a predictive biomarker in MM patients treated with cisplatin-based chemotherapy. In the future, it could help improve treatment stratification and potentially contribute to better treatment outcomes in MM.

## Figures and Tables

**Table 1:** Clinical characteristics of malignant mesothelioma patients.

		All Patients (N = 265)	Patients Treated with Cisplatin (N = 239)
Characteristic	Category/Unit	N (%)	N (%)
Sex	Male	197 (74.3)	179 (74.9)
	Female	68 (25.7)	60 (25.1)
Age	Years, median (25–75%)	66 (59–72)	66 (59–72)
Stage	1	17 (7.2)	15 (7.1)
	2	60 (25.5)	53 (25.1)
	3	85 (36.2)	77 (36.5)
	4	72 (30.6)	65 (30.8)
	Not determined	1 (0.4)	1 (0.5)
Location	Pleura	235 (88.7)	211 (88.3)
	Peritoneum	30 (11.3)	28 (11.7)
Histological type	Epithelioid	204 (77.0)	186 (77.8)
	Biphasic	23 (8.7)	21 (8.8)
	Sarcomatoid	26 (9.8)	23 (9.6)
	Not determined	12 (4.5)	9 (3.8)
ECOG performance status	0	15 (5.7)	15 (6.3)
	1	137 (51.7)	129 (54.0)
	2	106 (40.0)	92 (38.5)
	3	7 (2.6)	3 (1.3)
Asbestos exposure	No	66 (25.0) {1}	60 (25.2) {1}
	Yes	198 (75.0)	178 (74.8)
Smoking	No	146 (55.7) {3}	132 (55.7) {2}
	Yes	116 (44.3)	105 (44.3)
CRP	mg/L, median (25–75%)	23 (9–68.8) {29}	21 (8–61) {20}
LDH	µkat/L, median (25–75%)	2.72 (2.28–3.19) {28}	2.69 (2.26–3.17) {20}
Pain	No	109 (42.6) {9}	101 (43.5) {7}
	Yes	147 (57.4)	131 (56.5)
Weight loss	No	87 (34.7) {14}	77 (34.1) {13}
	Yes	164 (65.3)	149 (65.9)
Chemotherapy regimen	No	16 (6.0)	0 (0.0)
	Gemcitabine/cisplatin	154 (58.1)	154 (64.4)
	Pemetrexed/cisplatin	85 (32.1)	85 (35.6)
	Other	10 (3.8)	0 (0.0)
Disease progression	No	27 (10.2)	25 (10.5)
	Yes	238 (89.8)	214 (89.5)
Death	No	71 (26.8)	67 (28.0)
	Yes	194 (73.2)	172 (72.2)
PFS	Months, median (25–75%)	10.0 (6.3–16.2) **	8.2 (5.3–13.8) ***
OS	Months, median (25–75%)	19.1 (10.0–29.4) **	9.5 (18.1–28.4) ***
Follow-up	Months, median (25–75%)	45.5 (22.8–78.7) **	20.2 (44.4–75.5) ***
Calretinin concentration *	Ng/mL, median (25–75%)	0.52 (0.22–1.43)	0.51 (0.22–1.37)

The number of missing data is presented in {} brackets. * Data available for 146 patients in the whole cohort and 130 patients treated with cisplatin; ** survival and follow-up from diagnosis; *** survival and follow-up from the beginning of chemotherapy. CRP, C reactive protein, ECOG, Eastern Cooperative Oncology Group, LDH, lactate dehydrogenase, OS, overall survival, PFS, progression-free survival.

**Table 2:** Association of selected polymorphisms with progression-free survival (PFS) and overall survival (OS) from diagnosis of MM patients.

		PFS					OS				
SNP	Genotype	Median (25–75%)	HR (95% CI)	*p*	HR (95% CI)_adj1_	P_adj1_	Median (25–75%)	HR (95% CI)	*p*	HR (95% CI)_adj2_	P_adj2_
*CALB2* rs1862818	CC	9.8 (5.9–14.5)	Reference		Reference		16.7 (9.4–28.3)	Reference		Reference	
	CT	9.9 (6.6–16.9)	0.88 (0.67–1.15)	0.333	0.82 (0.60–1.11)	0.199	21.2 (11.3–35.1)	0.78 (0.58–1.05)	0.099	0.79 (0.57–1.09)	0.156
	TT	11.5 (6.6–20.5)	0.81 (0.52–1.27)	0.364	0.74 (0.45–1.21)	0.233	24.4 (12.5–27.2)	0.84 (0.52–1.37)	0.480	0.85 (0.50–1.44)	0.545
	CT + TT	10.0 (6.6–17.4)	0.86 (0.67–1.11)	0.256	0.80 (0.60–1.07)	0.130	22.0 (11.5–33.7)	0.79 (0.59–1.05)	0.099	0.80 (0.59–1.09)	0.157
*CALB2* rs889704	CC	10.0 (6.6–16.9)	Reference		Reference		18.5 (10.1–30.3)	Reference		Reference	
	CA	10.0 (6.0–12.6)	1.14 (0.83–1.56)	0.415	1.03 (0.72–1.46)	0.890	20.3 (9.5–28.6)	1.05 (0.73–1.51)	0.777	1.14 (0.78–1.66)	0.501
	AA	8.7 (4.8–8.7)	1.76 (0.43–7.13)	0.43	2.60 (0.63–10.68)	0.185	16.2 (6.1–16.2)	2.17 (0.53–8.81)	0.279	3.59 (0.86–14.96)	0.079
	CA + AA	9.7 (6.0–12.6)	1.15 (0.85–1.57)	0.362	1.06 (0.75–1.49)	0.762	20.3 (9.4–28.6)	1.08 (0.76–1.54)	0.665	1.18 (0.81–1.71)	0.381
*CALB2* rs8063760	CC	10.2 (6.6–17.1)	Reference		Reference		19.4 (10.0–29)	Reference		Reference	
	CT	9.4 (6.2–14.3)	1.07 (0.81–1.41)	0.622	1.26 (0.93–1.71)	0.143	19.3 (11.6–32.5)	0.88 (0.65–1.21)	0.433	0.98 (0.70–1.38)	0.920
	TT	7.9 (4.8–14.3)	1.54 (0.90–2.63)	0.114	1.22 (0.67–2.24)	0.518	12.7 (7.1–24.7)	1.59 (0.90–2.83)	0.112	1.22 (0.66–2.25)	0.527
	CT + TT	9.4 (6.1–14.3)	1.13 (0.87–1.46)	0.371	1.25 (0.94–1.67)	0.128	18.2 (10.0–31.1)	0.96 (0.72–1.29)	0.783	1.02 (0.74–1.41)	0.895
*E2F2* rs2075995	CC	8.4 (5.5–15.0)	Reference		Reference		16.0 (8.3–25.7)	Reference		Reference	
	CA	11.0 (6.6–16.2)	0.82 (0.60–1.12)	0.205	1.00 (0.71–1.42)	0.989	20.7 (11.0–31.4)	0.73 (0.52–1.02)	0.066	0.95 (0.65–1.39)	0.805
	AA	9.9 (6.2–16.5)	0.93 (0.65–1.33)	0.695	1.04 (0.70–1.55)	0.846	18.1 (10.8–28.6)	0.80 (0.54–1.19)	0.268	1.03 (0.66–1.60)	0.886
	CA + AA	10.2 (6.4–16.5)	0.85 (0.64–1.14)	0.286	1.02 (0.73–1.41)	0.928	19.7 (10.8–31.2)	0.75 (0.55–1.03)	0.075	0.98 (0.68–1.40)	0.903
*MIR335* rs3807348	GG	9.4 (5.6–15.0)	Reference		Reference		16.1 (7.6–25.6)	Reference		Reference	
	GA	10.6 (7.3–17.9)	0.83 (0.61–1.13)	0.239	0.98 (0.68–1.40)	0.900	22.0 (11.8–35.1)	0.69 (0.49–0.98)	0.038	0.65 (0.45–0.95)	0.028
	AA	8.2 (5.5–13.4)	1.18 (0.83–1.68)	0.353	1.40 (0.92–2.11)	0.114	15.6 (8.3–26.2)	1.08 (0.74–1.57)	0.709	1.02 (0.67–1.55)	0.914
	GA + AA	10.0 (6.6–16.3)	0.93 (0.69–1.24)	0.614	1.08 (0.77–1.52)	0.650	19.7 (11.0–31.2)	0.81 (0.59–1.11)	0.184	0.76 (0.53–1.08)	0.129
*NRF1* rs13241028	TT	10.7 (6.8–15.0)	Reference		Reference		18.2 (11.8–27.8)	Reference		Reference	
	TC	7.5 (5.6–16.3)	1.13 (0.86–1.49)	0.365	1.23 (0.92–1.67)	0.166	19.3 (7.3–33.7)	0.94 (0.69–1.26)	0.668	1.04 (0.75–1.45)	0.794
	CC	9.9 (5.5–14.1)	0.88 (0.47–1.62)	0.672	0.70 (0.28–1.77)	0.449	29.4 (9.6–*)	0.58 (0.27–1.25)	0.164	0.72 (0.29–1.81)	0.489
	TC + CC	8.0 (5.6–16.3)	1.10 (0.84–1.43)	0.490	1.19 (0.88–1.59)	0.257	19.3 (8.0–35.1)	0.89 (0.66–1.19)	0.416	1.01 (0.74–1.39)	0.942
*SEPTIN7* rs3801339	TT	11.6 (7.9–19.4)	Reference		Reference		22.0 (11.5–28.6)	Reference		Reference	
	TC	8.8 (6.0–16.3)	1.27 (0.90–1.80)	0.176	1.28 (0.88–1.88)	0.203	15.6 (10.0–29.0)	1.08 (0.74–1.56)	0.702	1.21 (0.81–1.80)	0.361
	CC	9.7 (6.1–14.7)	1.47 (1.02–2.13)	0.039	1.76 (1.17–2.64)	0.007	20.6 (9.6–31.4)	09.5 (0.64–1.42)	0.796	1.11 (0.72–1.71)	0.630
	TC + CC	9.4 (6.0–14.9)	1.35 (0.97–1.86)	0.074	1.44 (1.01–2.06)	0.046	17.5 (9.6–30.0)	1.02 (0.72–1.45)	0.905	1.17 (0.80–1.69)	0.421

Adj1: adjusted for asbestos exposure, smoking, weight loss, C-reactive protein, and histological type; Adj2: adjusted for age, asbestos exposure, smoking, C-reactive protein, and histological type. * not yet reached. Cl, confidence interval, HR, hazard ratio, OS, overall survival, PFS, progression-free survival, SNP, single-nucleotide polymorphism.

**Table 3:** Association of serum calretinin concentration with disease control rate, progression-free (PFS), and overall survival (OS) from the beginning of chemotherapy in MM patients treated with cisplatin-based chemotherapy.

	Disease Control Rate				PFS			OS		
Calretinin Concentration	CR + PR + SD	PD	OR (95% CI)	*p*	Median (25–75%)	HR (95% CI)	*p*	Median (25–75%)	HR (95% CI)	*p*
Serum concentration (ng/mL)	0.45 (0.21–1.07) *	1.68 (0.58–4.20) *	2.18 (1.51–3.14)	<0.001	/	1.21 (1.05–1.40)	0.010	/	1.23 (1.04–1.45)	0.013
<0.51 ng/mL, N (%) **	57 (91.9)	5 (8.1)	Ref.		7.9 (5.5–13.1)	Ref.		21.0 (10.1–42.5)	Ref.	
>0.51 ng/mL, N (%) **	48 (76.2)	15 (23.8)	3.56 (1.21–10.52)	0.021	7.0 (5.5–10.3)	1.38 (0.95–2.01)	0.090	15.0 (9.2–22.1)	1.6 (0.95–2.26)	0.085
<0.89 ng/mL, N (%) ***	75 (93.8)	5 (6.3)	Ref.		9.3 (6.1–13.9)	Ref.		21.4 (11.4–42.5)	Ref.	
>0.89 ng/mL, N (%) ***	30 (66.7)	15 (33.3)	7.50 (2.50–22.47)	<0.001	6.4 (4.2–8.8)	1.88 (1.28–2.77)	0.001	12.1 (8.3–19.6)	1.91 (1.22–2.97)	0.004

* Median (25–75%); ** divided based on median calretinin concentration; *** divided based on the cutoff value determined by ROC curve analysis. CR, complete response, PD, disease progression, PR, partial response, SD, stable disease.

**Table 4:** Association of selected polymorphisms with response rate in MM patients treated with cisplatin-based chemotherapy.

SNP	Genotype	SD + PD N (%)	PR + CR N (%)	OR (95% CI)	*p*	OR (95% CI)_adj_	P_adj_
*CALB2* rs1862818	CC	79 (71.8)	31 (28.2)	Reference		Reference	
	CT	58 (59.2)	40 (40.8)	1.76 (0.99–3.13)	0.056	1.69 (0.87–3.29)	0.121
	TT	14 (60.9)	9 (39.1)	1.64 (0.64–4.17)	0.301	1.31 (0.47–3.65)	0.607
	CT + TT	72 (59.5)	49 (40.5)	1.73 (1.00–3.01)	0.050	1.60 (0.85–3.01)	0.142
*CALB2* rs889704	CC	113 (63.5)	65 (36.5)	Reference		Reference	
	CA	35 (70.0)	15 (30.0)	0.75 (0.38–1.47)	0.395	0.76 (0.34–1.72)	0.511
	AA	3 (100)	0 (0)	/	0.306	/	/
	CA + AA	38 (71.7)	15 (28.3)	0.69 (0.35–1.34)	0.271	0.65 (0.29–1.45)	0.294
*CALB2* rs8063760	CC	87 (62.6)	52 (37.4)	Reference		Reference	
	CT	51 (65.4)	27 (34.6)	0.89 (0.50–1.58)	0.681	0.86 (0.45–1.67)	0.664
	TT	13 (92.9)	1 (7.1)	0.13 (0.02–1.01)	0.051	0.17 (0.02–1.46)	0.107
	CT + TT	64 (69.6)	28 (30.4)	0.73 (0.42–1.28)	0.276	0.74 (0.39–1.41)	0.361
*E2F2* rs2075995	CC	40 (70.2)	17 (29.8)	Reference		Reference	
	CA	76 (64.4)	42 (35.6)	1.30 (0.66–2.57)	0.450	0.84 (0.39–1.82)	0.657
	AA	35 (62.5)	21 (37.5)	1.41 (0.64–3.09)	0.389	1.51 (0.63–3.63)	0.355
	CA + AA	111 (63.8)	63 (36.2)	1.34 (0.70–2.55)	0.380	1.02 (0.50–2.11)	0.951
*MIR335* rs3807348	GG	45 (78.9)	12 (21.1)	Reference		Reference	
	GA	64 (55.7)	51 (44.3)	2.99 (1.43–6.23)	0.004	3.32 (1.40–7.89)	0.007
	AA	42 (71.2)	17 (28.8)	1.52 (0.65–3.55)	0.336	1.65 (0.61–4.49)	0.324
	GA + AA	106 (60.9)	68 (39.1)	2.41 (1.19–4.87)	0.015	2.69 (1.17–6.18)	0.020
*NRF1* rs13241028	TT	88 (63.3)	51 (36.7)	Reference		Reference	
	TC	57 (70.4)	24 (29.6)	0.73 (0.40–1.31)	0.287	0.64 (0.33–1.23)	0.182
	CC	6 (54.5)	5 (45.5)	1.44 (0.42–4.95)	0.565	1.89 (0.33–10.7)	0.473
	TC + CC	63 (68.5)	29 (31.5)	0.79 (0.45–1.39)	0.419	0.70 (0.37–1.32)	0.270
*SEPTIN7* rs3801339	TT	29 (60.4)	19 (39.6)	Reference		Reference	
	TC	73 (64.6)	40 (35.4)	0.84 (0.42–1.68)	0.614	0.93 (0.41–2.09)	0.860
	CC	49 (70.0)	21 (30.0)	0.65 (0.30–1.42)	0.281	0.56 (0.23–1.38)	0.209
	TC + CC	122 (66.7)	61 (33.3)	0.76 (0.40–1.47)	0.419	0.77 (0.36–1.66)	0.506

Adj: adjusted for smoking, CRP, and weight loss. Cl, confidence interval, CR, complete response, OR, odds ratio, PD, disease progression, PR, partial response, SD, stable disease, SNP, single-nucleotide polymorphism.

## Data Availability

All the data are presented within the article and Supplementary Materials. Any additional information is available upon request from the corresponding author.

## Supplementary Materials

The following supporting information can be downloaded at https://www.mdpi.com/article/10.3390/ijms25010190/s1.
