# Supplementary material for: Serum Calretinin and Genetic Variability as a Prognostic and Predictive Factor in Malignant Mesothelioma
Source: Int J Mol Sci. 2023 Dec 22;25(1):190. doi: 10.3390/ijms25010190 (PMC10778798; doi:10.3390/ijms25010190)
Supplement: Supplementary file 1 [file ijms-25-00190-s001.zip › ijms-2765815-supplementary.pdf]

Supplementary Materials

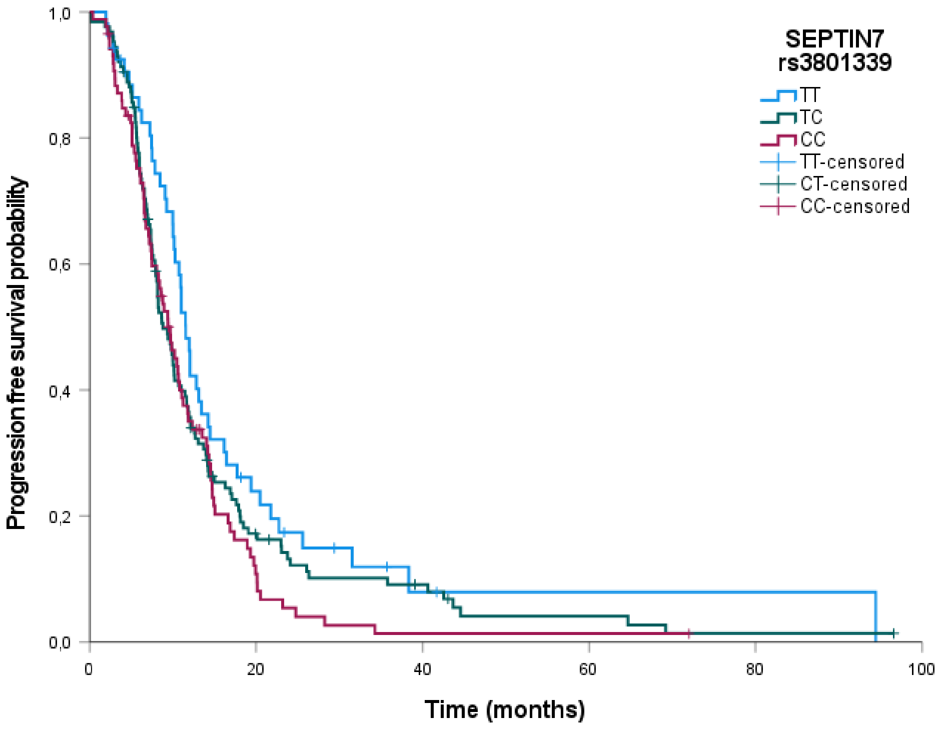

Figure S1. Association of *SEPTIN7* rs3801339 with progression free survival from diagnosis in malignant mesothelioma patients.

Table S1. Genotype frequencies of investigated single nucleotide polymorphisms.

Gene	SNP	Nucleotide or amino acid change	Genotype	N (%)	VAF	pHWE
CALB2	rs1862818	c.-828C>T	CC	132 (49.8)	0.296	0.826
			CT	109 (41.1)		
			TT	24 (9.1)		
CALB2	rs889704	c.-634C>A	CC	203 (76.6)	0.123	0.574
			CA	59 (22.3)		
			AA	3 (1.1)		
CALB2	rs8063760	c.*138T>C	CC	162 (61.1)	0.223	0.508
			CT	88 (33.2)		
			TT	15 (5.7)		
E2F2	rs2075995	c.678C>A, p.Gln226His	CC	68 (25.7)	0.492	0.948
			CA	133 (50.2)		
			AA	64 (24.2)		
MIR335	rs3807348	g.130496266G>A	GG	67 (25.3)	0.504	0.668
			GA	129 (48.7)		
			AA	69 (26.0)		
NRF1	rs13241028	c.*1321T>C	TT	161 (60.8)	0.219	0.803
			TC	92 (34.7)		
			CC	12 (4.5)		
SEPTIN7	rs3801339	c.1168-4451T>C	TT	53 (20.0)	0.562	0.579
			TC	126 (47.5)		
			CC	86 (32.5)		

HWE, Hardy–Weinberg equilibrium, SNP, single-nucleotide polymorphism, VAF, variant allele frequency

Table S2. Association of serum calretinin concentration with disease control rate, progression-free (PFS) and overall survival (OS) from the beginning of chemotherapy in MM patients treated with cisplatin-based chemotherapy: adjustment for clinical parameters.

	Disease control rate		PFS		OS	
**Calretinin concentration**	**OR (95% CI)$_{adj1}$**	**P$_{adj1}$**	**HR (95% CI)$_{adj2}$**	**P$_{adj2}$**	**HR (95% CI)$_{adj3}$**	**P$_{adj3}$**
Serum concentration (ng/ml)	1.95 (1.23-3.10)	0.004	1.14 (0.93-1.39)	0.206	1.10 (0.89-1.37)	0.378
<0.51 ng/ml, N (%)*	Reference		Reference		Reference	
>0.51 ng/ml, N (%)*	2.49 (0.68-9.07)	0.168	1.08 (0.71-1.66)	0.709	0.95 (0.58-1.58)	0.852
<0.89 ng/ml, N (%)**	Reference		Reference		Reference	
>0.89 ng/ml, N (%)**	5.10 (1.36-19.10)	0.016	1.90 (1.20-3.00)	0.006	1.75 (1.00-3.03)	0.049

*based on median calretinin concentration; **based on cutoff value determined in ROC curve analysis

Adj1: adjusted for CRP in chemotherapy type; Adj2: adjusted for asbestos exposure, smoking, weight loss, CRP and histological type; Adj3: adjusted for smoking, CRP and histological type

Table S3. Association of selected polymorphisms with progression-free (PFS) and overall survival (OS) from the beginning of chemotherapy in MM patients treated with cisplatin-based chemotherapy.

		PFS					OS				
**SNP**	**Genotype**	**Median (25%-75%)**	**HR (95% CI)**	**P**	**HR (95% CI)$_{adj1}$**	**P$_{adj1}$**	**Median (25%-75%)**	**HR (95% CI)**	**P**	**HR (95% CI)$_{adj2}$**	**P$_{adj2}$**
CALB2 rs1862818	CC	7.8 (5.2-12.7)	Reference		Reference		16.0 (8.7-26.5)	Reference		Reference	
	CT	7.9 (5.6-13.8)	0.86 (0.65-1.15)	0.316	0.90 (0.65-1.24)	0.512	19.2 (10.6-32.5)	0.75 (0.54-1.03)	0.078	0.78 (0.55-1.09)	0.144
	TT	9.1 (5.4-18.5)	0.80 (0.51-1.27)	0.350	0.74 (0.45-1.22)	0.235	22.1 (10.6-26.7)	0.80 (0.48-1.34)	0.400	0.85 (0.50-1.44)	0.538
	CT+TT	8.9 (5.5-14.1)	0.85 (0.65-1.11)	0.241	0.86 (0.63-1.16)	0.318	20.5 (10.6-29.8)	0.76 (0.56-1.03)	0.075	0.79 (0.57-1.09)	0.149
CALB2 rs889704	CC	8.5 (5.4-14.1)	Reference		Reference		18.3 (9.9-28.7)	Reference		Reference	
	CA	7.7 (5.1-10.5)	1.22 (0.88-1.70)	0.231	1.12 (0.77-1.61)	0.557	16.0 (8.7-28.4)	1.19 (0.81-1.74)	0.372	1.22 (0.83-1.80)	0.320
	AA	6.4 (5.5-*)	1.51 (0.37-6.15)	0.563	2.29 (0.56-9.43)	0.251	14.0 (6.8-14.0)	2.29 (0.56-9.35)	0.248	4.49 (1.08-18.68)	0.039
	CA+AA	7.7 (5.2-10.5)	1.23 (0.89-1.70)	0.206	1.14 (0.80-1.64)	0.461	15.6 (8.7-28.4)	1.22 (0.84-1.77)	0.294	1.27 (0.87-1.86)	0.219
CALB2 rs8063760	CC	8.6 (5.2-15.0)	Reference		Reference		18.2 (9.4-26.8)	Reference		Reference	
	CT	7.9 (5.3-13.0)	1.01 (0.75-1.34)	0.960	1.17 (0.85-1.60)	0.346	18.5 (10.1-30.2)	0.86 (0.62-1.19)	0.356	0.93 (0.65-1.32)	0.681
	TT	6.7 (4.8-9.5)	1.57 (0.90-2.74)	0.111	1.12 (0.60-2.07)	0.723	12.0 (6.8-21.1)	1.71 (0.94-3.12)	0.080	1.47 (0.80-2.71)	0.212
	CT+TT	7.8 (5.3-11.9)	1.07 (0.81-1.41)	0.633	1.16 (0.86-1.56)	0.338	18.1 (9.9-29.6)	0.94 (0.69-1.28)	0.704	1.01 (0.73-1.40)	0.960
E2F2 rs2075995	CC	7.8 (5.3-13.6)	Reference		Reference		14.9 (9.1-23.6)	Reference		Reference	
	CA	8.6 (5.4-13.9)	0.83 (0.60-1.15)	0.263	1.03 (0.71-1.48)	0.895	19.2 (9.5-29.7)	0.71 (0.50-1.02)	0.063	0.90 (0.60-1.35)	0.626
	AA	8.5 (5.3-13.8)	0.90 (0.62-1.31)	0.576	1.07 (0.70-1.64)	0.746	18.3 (10.1-28.4)	0.78 (0.51-1.18)	0.244	1.05 (0.66-1.66)	0.848
	CA+AA	8.6 (5.3-13.9)	0.85 (0.63-1.16)	0.307	1.04 (0.74-1.47)	0.823	18.5 (9.8-29.6)	0.73 (0.52-1.03)	0.071	0.95 (0.65-1.39)	0.786
	GG	8.6 (5.3-14.1)	Reference		Reference		15.6 (8.7-23.9)	Reference		Reference	

MIR335 rs3807348	GA	8.5 (6.0-13.9)	0.99 (0.71-1.37)	0.937	1.24 (0.84-1.81)	0.275	18.3 (10.1-29.7)	0.81 (0.56-1.18)	0.278	0.71 (0.48-1.06)	0.096
	AA	6.9 (4.6-11.7)	1.24 (0.85-1.80)	0.273	1.47 (0.95-2.27)	0.085	16.0 (8.7-26.7)	1.07 (0.71-1.62)	0.741	0.96 (0.62-1.50)	0.874
	GA+AA	7.9 (5.4-13.1)	1.06 (0.78-1.45)	0.716	1.30 (0.90-1.88)	0.156	18.2 (9.7-28.7)	0.89 (0.63-1.26)	0.524	0.79 (0.54-1.15)	0.216
NRF1 rs13241028	TT	9.1 (5.6-13.8)	Reference		Reference		18 (9.9-25.4)	Reference		Reference	
	TC	7.0 (4.4-13.0)	1.10 (0.83-1.47)	0.500	1.24 (0.90-1.70)	0.189	18.3 (8.3-35.8)	0.90 (0.65-1.24)	0.508	1.00 (0.71-1.40)	0.991
	CC	9.2 (4.8-31.3)	0.81 (0.42-1.55)	0.521	0.68 (0.27-1.74)	0.422	28.8 (8.7-*)	0.51 (0.22-1.16)	0.110	0.69 (0.28-1.71)	0.421
	TC+CC	7.1 (4.4-13.0)	1.06 (0.80-1.40)	0.684	1.19 (0.87-1.62)	0.289	18.5 (8.7-35.8)	0.84 (0.62-1.15)	0.272	0.96 (0.69-1.34)	0.829
SEPTIN7 rs3801339	TT	9.2 (6.4-12.8)	Reference		Reference		18.3 (9.1-22.7)	Reference		Reference	
	TC	7.7 (5.2-14.7)	1.02 (0.72-1.46)	0.896	0.89 (0.60-1.33)	0.571	15.0 (9.5-28.7)	0.82 (0.56-1.21)	0.325	0.88 (0.59-1.32)	0.533
	CC	7.6 (4.2-13.6)	1.20 (0.82-1.77)	0.343	1.34 (0.88-2.04)	0.167	21.8 (9.9-29.7)	0.69 (0.45-1.06)	0.092	0.86 (0.55-1.35)	0.517
	TC+CC	7.7 (5.2-13.9)	1.09 (0.78-1.52)	0.619	1.04 (0.72-1.51)	0.828	18.1 (9.7-29.6)	0.77 (0.54-1.11)	0.160	0.87 (0.60-1.28)	0.485

Adj1: adjusted for asbestos exposure, smoking, weight loss, CRP and histological type; Adj2: adjusted for smoking, CRP and histological type
*not yet reached
CI, confidence interval, HR, hazard ratio, OS, overall survival, PFS, progression-free survival, SNP, single nucleotide polymorphism
